# T169. COGNITIVE INSIGHT AND CORTICAL THICKNESS IN SCHIZOPHRENIA

**DOI:** 10.1093/schbul/sby016.445

**Published:** 2018-04-01

**Authors:** Arpitha Jacob, Mariamma Philip, Rose Bharath, Shivarama Varambally, Ganesan Venkatasubramanian, Gopikrishna Deshpande, Naren Rao, Ayushi Shukla

**Affiliations:** 1 National Institute of Mental Health and Neurosciences; 2 Auburn University

## Abstract

**Background:**

Diminished cognitive insight is exhibited by substantial proportion of patients suffering from schizophrenia and is an important determinant of poor treatment adherence. While the clinical correlates of cognitive insight are well examined the neural correlates of cognitive insight is less explored. We examined relation between cortical thickness and cognitive insight in schizophrenia patients.

**Methods:**

We examined 37 schizophrenia patients in comparison with 19 healthy volunteers. We measured cortical thickness using a high resolution anatomical magnetic resonance image and cognitive insight using Beck’s Cognitive Insight Scale (BCIS). We measured the difference between schizophrenia patients and healthy volunteers using Analysis of covariance and relation between cortical thickness and BCIS scores in schizophrenia patients using stepwise regression analysis.

**Results:**

Patients had significantly thinner cortices than healthy volunteers in orbitofrontal cortex, superior temporal gyrus, occipital cortex, dorsomedial prefrontal cortex and posterior cingulate cortex. Significant positive correlations were found between self-reflection and cortical thickness in posterior cingulate cortex, dorso-medial frontal gyrus, occipital lobe. Significant negative correlations were observed between self-certainty scores and bilateral Posterior cingulate and orbitofrontal cortex.

**Discussion:**

We found significant differences in cortical thickness between SCZ and HV in brain regions implicated in cognitive insight. Our findings also suggest higher self-certainty to be associated with thinner cortices in bilateral PCC and OFC. Significant relations between cortical thickness and cortical midline structures supports the critical role of these self-evaluative brain regions in cognitive insight in schizophrenia.

